# Impact of a posttraumatic cerebral infarction on outcome in patients with TBI: the Italian multicenter cohort INCEPT study

**DOI:** 10.1186/s13054-020-2746-5

**Published:** 2020-02-03

**Authors:** Nicola Latronico, Simone Piva, Nazzareno Fagoni, Lorenzo Pinelli, Michele Frigerio, Davide Tintori, Maurizio Berardino, Andrea Bottazzi, Livio Carnevale, Tiziana Casalicchio, Carlo Alberto Castioni, Simona Cavallo, Davide Cerasti, Giuseppe Citerio, Marco Fontanella, Serena Galiberti, Alan Girardini, Paolo Gritti, Ornella Manara, Paolo Maremmani, Roberta Mazzani, Giuseppe Natalini, Mirko Patassini, Maria Elena Perna, Ilaria Pesaresi, Danila Katia Radolovich, Maurizio Saini, Roberto Stefini, Cosetta Minelli, Roberto Gasparotti, Francesco A. Rasulo

**Affiliations:** 1 Department of Medical and Surgical Specialties, Radiological Sciences and Public Health, University of Brescia, Brescia, Italy; 2 Department of Anesthesia, Intensive Care and Emergency, ASST Spedali Civili University Hospital, Piazzale Ospedali Civili 1, 25121 Brescia, Italy; 3 Department of Molecular and Translational Medicine, University of Brescia, Brescia, Italy; 4 Department of Radiology, Neuroradiology Unit, ASST Spedali Civili University Hospital, Brescia, Italy; 5 Anesthesia and Intensive Care Unit, AOU Città della Salute e della Scienza, Presidio CTO, Turin, Italy; 6 Department of Anesthesia and Critical Care Medicine, IRCCS Policlinico San Matteo Pavia, Pavia, Italy; 7 Department of Anestesiology and Intensive Care Medicine, S. Giovanni Bosco Hospital, ASLTO2, Turin, Italy; 8 Department of Neuroradiology, Ospedale Maggiore, University of Parma, Parma, Italy; 9 School of Medicine and Surgery, University of Milan-Bicocca, Milan, Italy; 10 Department of Anesthesia and Critical Care Medicine, Unit of Neurointensive Care Medicine, ASST-Monza, Monza, Italy; 11 Division of Neurosurgery, Department of Medical and Surgical Specialties, Radiological Sciences and Public Health, University of Brescia, Brescia, Italy; 12 Department of Anesthesia and Critical Care Medicine, Azienda Ospedaliera Universitaria Pisana, Pisa, Italy; 13 Department of Anesthesia and Intensive Care, Fondazione Poliambulanza Hospital, Brescia, Italy; 14 Department of Anesthesia and Critical Care Medicine, Papa Giovanni XXIII Hospital, ASST Bergamo, Bergamo, Italy; 15 Department of Neuroradiology, Papa Giovanni XXIII Hospital, ASST Bergamo, Bergamo, Italy; 16 Department of Anesthesiology, Critical Care and Pain Medicine, Maggiore University Hospital, Parma, Italy; 17 Department of Radiology, Section of Neuroradiology, ASST-Monza, Monza, Italy; 18 Department of Radiology, S. Giovanni Bosco Hospital, ASLTO2, Turin, Italy; 19 Department of Diagnosis and Imaging, Neuroradiology, Azienda Ospedaliera Universitaria Pisana, Pisa, Italy; 20 Department of Neuroscience, Legnano Hospital, Milan, Italy; 21 The National Heart and Lung Institute (NHLI), Imperial College London, London, UK

**Keywords:** Traumatic brain injury, Posttraumatic cerebral infarction, Long term outcome, Disability

## Abstract

**Background:**

Post-traumatic cerebral infarction (PTCI) is common after traumatic brain injury (TBI). It is unclear what the occurrence of a PTCI is, how it impacts the long-term outcome, and whether it adds incremental prognostic value to established outcome predictors.

**Methods:**

This was a prospective multicenter cohort study of moderate and severe TBI patients. The primary objective was to evaluate if PTCI was an independent risk factor for the 6-month outcome assessed with the Glasgow Outcome Scale (GOS). We also assessed the PTCI occurrence and if it adds incremental value to the International Mission for Prognosis and Clinical Trial design in TBI (IMPACT) core and extended models.

**Results:**

We enrolled 143 patients, of whom 47 (32.9%) developed a PTCI. In the multiple ordered logistic regression, PTCI was retained in both the core and extended IMPACT models as an independent predictor of the GOS. The predictive performances increased significantly when PTCI was added to the IMPACT core model (AUC = 0.73, 95% C.I. 0.66–0.82; increased to AUC = 0.79, 95% CI 0.71–0.83, *p* = 0.0007) and extended model (AUC = 0.74, 95% C.I. 0.65–0.81 increased to AUC = 0.80, 95% C.I. 0.69–0.85; *p* = 0.00008). Patients with PTCI showed higher ICU mortality and 6-month mortality, whereas hospital mortality did not differ between the two groups.

**Conclusions:**

PTCI is a common complication in patients suffering from a moderate or severe TBI and is an independent risk factor for long-term disability. The addition of PTCI to the IMPACT core and extended predictive models significantly increased their performance in predicting the GOS.

**Trial registration:**

The present study was registered in ClinicalTrial.gov with the ID number NCT02430324.

## Background

A traumatic brain injury (TBI) is a leading cause of mortality and morbidity mostly among young people; although, its incidence is increasing in older people, particularly in high-income countries [1]. The outcome of a TBI depends on several factors, including the patients’ characteristics, disease severity at admission, and complications arising during its clinical course. Multivariable prognostic models such as the International Mission for Prognosis and Clinical Trial design in TBI (IMPACT) have shown that most prognostic information is contained in a *core* set of three predictors: age, the Glasgow Coma Scale (GCS) motor score (GCSm), and pupillary reactivity [2]. IMPACT also provided an *extended prognostic model*, which adds the brain computed tomography (CT) classification and secondary cerebral insults, such as hypoxia and hypotension, to the core variables. Both core and extended IMPACT models focus on identifying prognostic factors at baseline and do not include predictors from the intensive care unit (ICU) stay [3]. Posttraumatic cerebral infarction (PTCI) is a common complication of a TBI in the acute stage of the disease. A PTCI is frequent in patients who die after a moderate or severe TBI, with a reported incidence in post-mortem studies up to 90%. The ante-mortem occurrence rate of a PTCI has been assessed in six single-center studies, of which only one was prospective, and the rate varied between 1.9% and 20.3% [4–9]. None of these studies investigated whether PTCI adds incremental value to current prognostic models. Therefore, we planned a multicenter, prospective observational cohort study in patients with a moderate or severe TBI to investigate: (1) the impact of PTCI on the 6-month outcome evaluated by the Glasgow Outcome Scale (GOS), (2) if the PTCI adds incremental value beyond that provided by the IMPACT prediction models on the GOS at 6 months, and (3) the occurrence of PTCI in the study population.

## Methods

In the present prospective observational cohort study (ClinicalTrials.gov Identifier: NCT02430324), we included all consecutive adult patients aged ≥ 16 years with a moderate (post-resuscitation GCS 12 to 9) or severe (post-resuscitation GCS 8 to 3) TBI who were admitted to the ICUs of nine Italian trauma centers from December 2009 to December 2012. Exclusion criteria were a history of cerebral ischemia, CT evidence of brain ischemia at admission, absence of invasive intracranial pressure (ICP) monitoring, and patients with a GCS score of 3 and unreactive pupils.

### Patient management

All patients were intubated, mechanically ventilated, underwent ICP and arterial blood pressure monitoring and ECG and were monitored for peripheral oxygen saturation and end-tidal CO_2_. Management was in accordance with international recommendations aimed at aggressively treating intracranial hypertension and rapidly correcting secondary cerebral insults [10].

### Ethics approval

The study was conducted in accordance with the Declaration of Helsinki and was approved by the local Ethics Committees of each participating center. Patients’ informed consent was waived due to the lack of definition of a legal representative of temporarily incapacitated adult patients in the Italian legislation [11]. The informed consent was obtained from the surviving patients as soon as they regained their mental competency. Family members received detailed information on the study scope and protocol. We followed the STROBE (Strengthening the Reporting of Observational Studies in Epidemiology) guidelines for reporting cohort studies [12].

### Data collection

Data on age, sex, GCS, the ICU and hospital length of stay (LOS), days of mechanical ventilation, type of surgery, major cardio-circulatory events (systemic hypotension, life-threatening cardiac arrhythmias, cardiac arrest), brain CT scans (see below), and GOS were prospectively collected. Several of these variables are risk factors for PTCI development, and their association with PTCI risk in this patient population will be investigated and reported in a separate article.

### Imaging

Posttraumatic brain CT findings were classified according to Marshall et al. [13, 14]. Brain CT scans were performed at hospital admission and then repeated within 24 h or within 12 h if the first CT scan had been obtained within 3 h after injury, in case of neurological deterioration or an increase in ICP [15–17]. A third CT scan might be scheduled on the 3rd day post-trauma [15]. Follow-up brain CTs were performed at the discretion of the attending physicians of each participating center.

The final diagnosis of PTCI with identification of the time of onset and the type of infarction (territorial cerebral infarction, watershed cerebral infarction, and non-territorial, non-watershed cerebral infarction, see below), as well as the presence of cerebral herniation, CT signs of intracranial hypertension, Marshall CT score, presence of subarachnoid hemorrhage (SAH) or epidural hematoma (EDH), and the midline shift was performed after central revision of the entire brain CT data set of all patients enrolled. Two senior neuro-radiologists (LP, MF) of the University of Brescia, who were blinded to each other’s diagnosis and to the patient’s outcome, assessed the brain CT based on DICOM (Digital Imaging and Communications in Medicine) scans of each patient in duplicate. Each neuroradiologist independently performed a “one shot” assessment of the entire neuroradiological history of each patient, with an immediate comparison of any questionable findings in a given CT scan to several previous and following exams, thus making the final diagnosis of cerebral infarction much more reliable. In case of discordance, an agreement was reached by a third neuro-radiologist (RG).

Only cerebral infarction that developed after trauma was considered; ischemic lesions identified on the first CT scan whose density remained unchanged during the neuroradiologic follow-up were considered old infarcts and ignored. Moreover, subtle brain CT hypodensities of uncertain classification due to indistinct margins and no clear mass effect at the first scan were diagnosed as PTCI if one or more of the following findings were present in the follow-up CT exams: (1) an increased hypodensity in the following 24–48 h, with progressive delineation of sharp margins; (2) a wedge-shaped lesion with clear effacement of the cerebral sulci when the lesion was cortical, often in a specific vascular territory of a major cerebral artery; (3) a progressive attenuation of the CT findings in the subacute phase of the ischemia (the so-called “fogging effect”). Conversely, subtle brain CT hypodensities suggesting PTCI in a given brain CT scan, which (1) faded away at the 24 h CT control, or (2) completely disappeared at longer CT follow-up, or (3) did not follow the expected CT changes for an ischemic lesion, were discarded as a non-ischemic lesion.

Following the definition used in our previous study [4], cerebral infarction was classified according to the following criteria [18–20]: (1) territorial cerebral infarction: well demarcated hypodense lesions within a defined cerebral vascular territory, involving the entire arterial territory (complete infarct) or only part of it (incomplete infarct); the vascular territories considered were the anterior cerebral artery (ACA), middle cerebral artery (MCA), posterior cerebral artery (PCA), lenticulostriate arteries (LSAs), anterior choroidal arteries, thalamo-perforating arteries (TPAs), basilar artery (BA), anterior–inferior cerebellar artery, superior cerebellar artery (SCA), and posterior–inferior cerebellar artery (PICA); (2) watershed cerebral infarction: well demarcated hypodense lesions positioned in boundary zones between the territories of the ACA, MCA, and PCA (superficial or leptomeningeal border zones) or in the terminal zones of the perforating arteries within the deep white matter (deep or medullary border zones); (3) non-territorial, non-watershed cerebral infarction: single or multiple hypodense lesions, unilateral, bilateral, or multifocal with marked borders without a precise localization in a vascular territory.

### Outcomes

The primary outcome was the GOS at 6 months (5 = good recovery; 4 = moderate disability; 3 = severe disability; 2 = vegetative state; 1 = death) [21]. In particular, we wanted to evaluate if a PTCI was an independent risk factor for the GOS and if it added incremental value in predicting long-term outcome compared with the IMPACT prediction model. The secondary outcomes were (1) the occurrence rate of PTCI in patients with a moderate or severe TBI and (2) the association of PTCI with hospital and ICU mortality, as well as the 6-month mortality. The occurrence rate was also calculated as the number of cases of PTCI during the observation period divided by the total number of patients enrolled in the study [22].

### Statistical analysis

Continuous variables are presented as the mean and standard deviation if normally distributed; while the median and interquartile range was used for non-normally distributed continuous variables and ordinal variables. Discrete variables are reported as the count and percentage. We did not have any missing data at baseline or follow-up (GOS at 6 months). Differences between patients with and without PTCI were tested using the *t*-test or Mann-Whitney *U* test, as appropriate, for continuous variables (age, ICU LOS, hospital LOS, days of mechanical ventilation), and the *χ*^*2*^ test for binary and categorical variables (secondary cerebral insults, coma, SAH or EDH, Marshall brain CT). We performed a sample size calculation for the association of PTCI with the dichotomized GOS at 6 months (unfavorable outcome, GOS ≤ 3). We estimated that 116 patients were needed to obtain a power of 80% and to detect a minimum odds ratio of 3, considering a prevalence of unfavorable outcome in patients without PTCI of 25% at a two-sided significance level of 0.05.

Ordered logistic regression (proportional odds logistic regression) was used to assess whether PTCI could predict the 6-month GOS (ordinal outcome) [23] using simple regression analysis and whether PTCI remained an independent predictor after adding the predictors from the IMPACT models using multiple regression analysis. In particular, we first assessed the association of the GOS with the variables included in the IMPACT core model (age, GCSm, and pupillary reactivity) and in the extended model (core variables plus Marshall CT classification scale, SAH or EDH, and secondary cerebral insults, in particular, hypoxia and hypotension). We then added PTCI to each model to evaluate its independent association with the GOS [3]. The proportionality assumption was checked for each selected predictor.

To assess the incremental predictive performance of the model when adding PTCI, we dichotomized the 6-month GOS into “unfavorable” (GOS ≤ 3) and “favorable” (GOS 4 and 5) outcomes, performed logistic regression analysis for the core and extended models with and without PTCI, and compared the area under the receiver operating characteristic curve (AUC) of the models with PTCI versus those without PTCI. The AUC varies between 0.5 (a noninformative model) and 1.0 (a perfect predictive model). Differences in the AUC between models were tested using the roc.test function in R (package pROC). To internally validate our model and to avoid over-optimism, we used a bootstrap procedure [24] using the “auc.adjust” R function. Finally, we repeated the AUC comparison using the AUC corrected for optimism. All statistical tests were two-tailed, and statistical significance was defined as *p* < 0.05. All statistical analyses were performed using R (3.0.3).

## Results

**Fig. 1:**
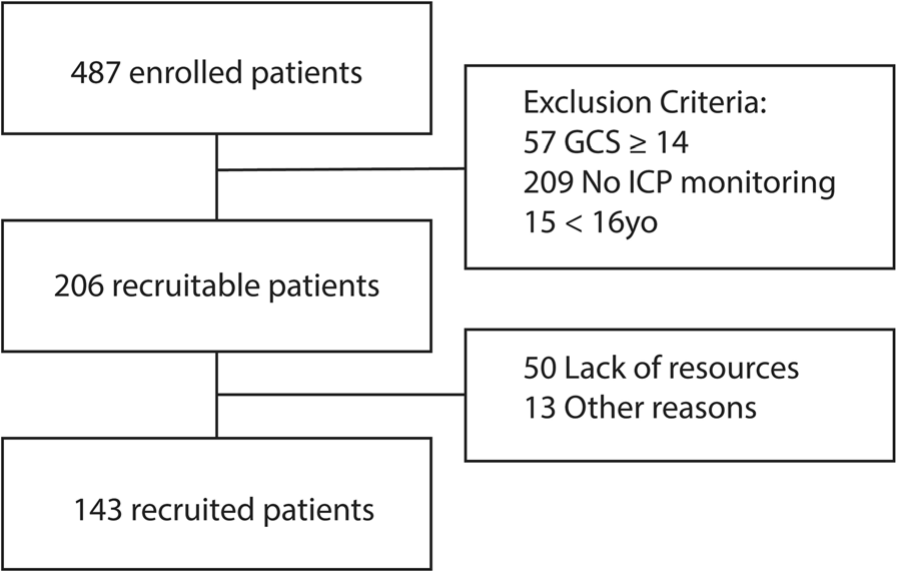
Study flow chart

**Fig. 2:**
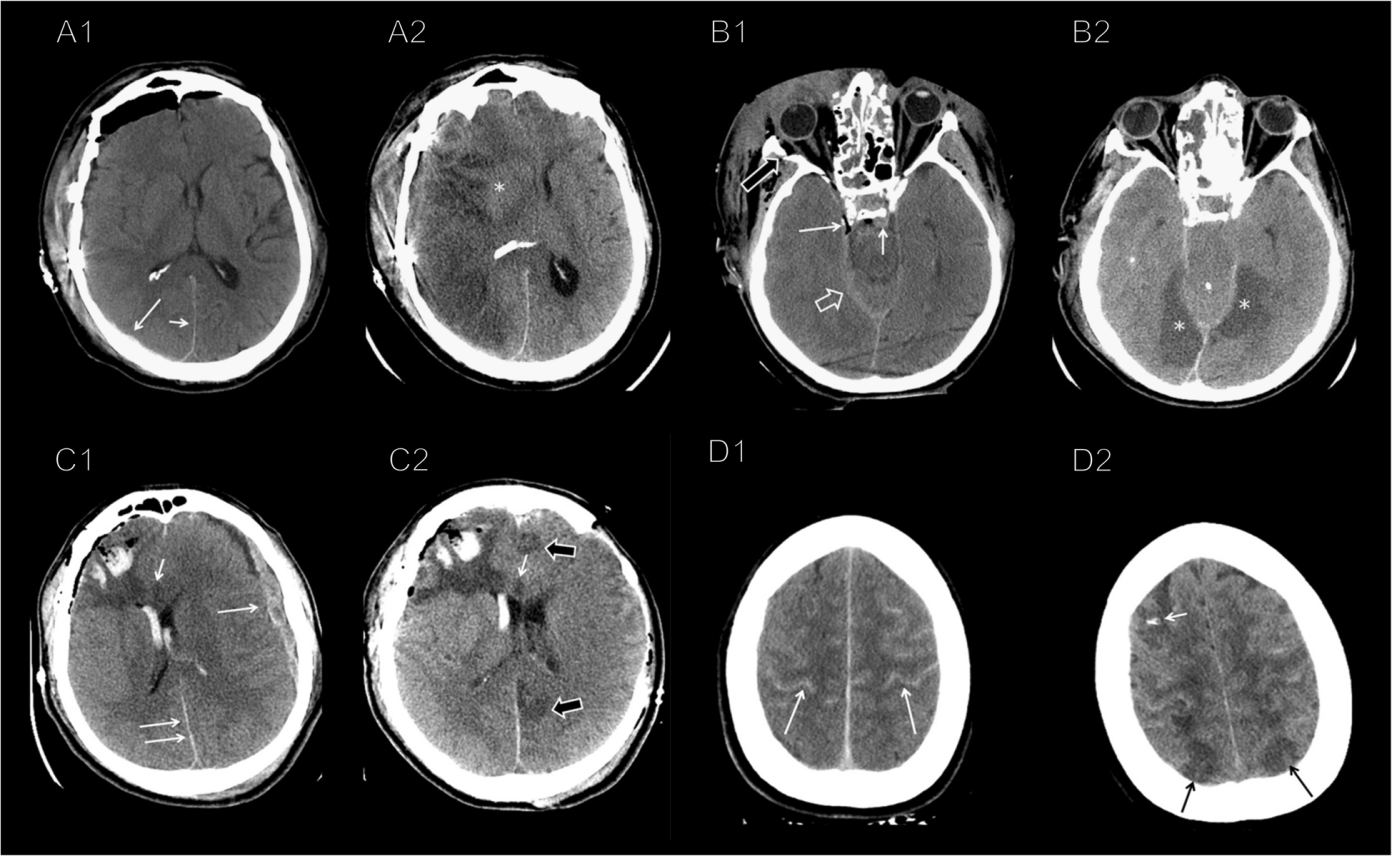
CT scan showing posttraumatic cerebral infarction (PTCI). **A1** MCA PTCI: acute parietal subdural hematoma on the right side (long arrow), extending to the falx (short arrow). **A2** CT scan 9 days later showed an *acute ischemic lesion in the superficial territory of the right MCA* (preserved right lenticular nucleus, white *). **B2** PCA PTCI: acute subdural hematoma along the right side of the tentorium (empty arrow), extra-axial blood in the prepontine cistern (short arrow), and small para sellar bubble air (long arrow) on admission brain CT. **B2** Brain CT scan at 15 days showed complete effacement of the basal cisterns and bilateral temporo-occipital hypodensities (*), consistent with *acute ischemic lesions in the territory of both PCA*. **C1** ACA PTCI: hemorrhagic contusions of the right frontal lobe mixed with air and perilesional vasogenic edema, intraventricular hemorrhage, a thick left frontoparietal acute subdural hematoma (long arrow) with midline shift to the right, and a thin acute subdural hematoma along the posterior falx (double arrows). **C2** Left frontoparietal craniectomy and hematoma evacuation showed multifocal hypodensities in the anterior and posterior portion of the left cingulate gyrus (white outlined arrows), consistent with *acute ischemic lesions in the territory of the left ACA*. The small hypodensity in the genu of the corpus callosum (short arrow), barely visible in the first exam, is consistent with a shear-strain injury. **D1** Superficial watershed PTCI: thick acute subdural hematomas along the whole tentorium and the left frontotemporal convexity. Diffuse subarachnoid hemorrhage is also visible at the vertex (long white arrows). **D2** Bilateral cortical hypodensities in the posterior parasagittal regions (black arrows), consistent with *acute watershed ischemia at the boundary zone between the MCA and ACA territories*. Note the probe for the intracranial pressure monitoring in the left frontal lobe (short white arrow)

During the study period, 487 patients with a TBI were admitted to the 9 participating ICUs with a final 143 patients (29.3%) enrolled in the study (Fig. 1). Of these, 47 (32.9%) patients developed a PTCI. There were no differences in patients with and without PTCI in terms of age, severity of the TBI, presence of SAH or EPH on the admission brain CT, hospital LOS, and days of mechanical ventilation (Table 1, Table 2). The incidences of intra-hospital hypotension and hypoxia, pupillary light reflex abnormalities, and evacuated mass lesions (defined according to Marshall brain CT classification) were higher in patients developing a PTCI (Table 1). A total of 94 cerebral infarctions developed in 47 patients, 81 were territorial (86.2%) and 8 were watershed (8.5%). Five infarctions could not be attributed either to territorial or watershed types. Territorial infarctions were in the area of the MCA (*n* = 17; 18.1%), ACA, (*n* = 18; 19.1%), PCA (*n* = 21; 22.3%), LSA (*n* = 8; 8.5%), TPA (*n* = 7; 7.4%), BA (*n* = 3; 3.2%), SCA (*n* = 3; 32%), PICA (*n* = 1; 1.1%), and anterior communicating artery (AcoA) (*n* = 3; 3.2%). Watershed infarctions were in the boundary zones (*n* = 3; 3.2%) and terminal zones (*n* = 5; 5.3%). The mean (SD) onset time of a PTCI was 6.2 (11.4) days with an early peak within 24 h (21 cases, 39.6%), a late peak between 3 and 7 days (14 cases, 26.4%), and 4 cases (7.6%) between 24 and 48 h, Fig. 2.

**Table 1:** Demographic and clinical data

Variables	PTCI (*n* = 47; 32.9%)	Non-PTCI (*n* = 96; 67.1%)	*p* value
Age, mean (SD) years	43.8 (17.2)	42.1 (18.4)	0.606
Sex, no. (%) males	38 (80.8%)	75 (78.1%)	0.141
Admission GCS, median (IQR)	6 (4–8)	7 (5–9)	0.704
Severe head trauma, no. (%)	37 (78.7%)	70 (72.9%)	0.452
Moderate head trauma, no. (%)	10 (21.3%)	26 (27.1%)	0.452
Secondary cerebral insults in the first 48 h, no. (%)			
Pre-hospital hypoxia	5 (10.6%)	7 (7.3%)	0.460
Intra-hospital hypoxia	7 (14.9%)	13 (13.5%)	0.048
Cardiac arrest	11 (23.4%)	1 (1.0%)	< 0.0001
Pre-hospital systemic hypotension	8 (17.0%)	39	0.021
Intra-hospital systemic hypotension	12 (25.5%)	35	0.274
Hyperthermia intra-H	13 (27.7%)	34	0.289
Pupillary light reflex, no. (%)			
Both present	30 (63.8%)	85 (88.5%)	0.002
One present	7 (14.9%)	3 (3.1%)	
Both absent	10 (21.3%)	8 (8.3%)	
SAH on admission brain CT, no. (%)	33 (70.2)	65 (67.7)	0.849
EDH on admission brain CT, no. (%)	15 (31.9)	27 (28.1)	0.697
Admission brain CT, Marshall CT classification, no. (%)			
Diffuse injury I	1 (2.3)	0 (0.0)	0.035
Diffuse injury II	10 (23.3)	42 (46.7)	
Diffuse injury III	3 (7.0)	4 (4.4)	
Diffuse injury IV	2 (4.7)	5 (5.6)	
Evacuated mass lesions	25 (58.1)	36 (40.0)	
Non-evacuated mass lesions	2 (4.7)	3 (3.3)	

Abbreviation: *GCS* Glasgow Coma Scale, *SAH* traumatic subarachnoid hemorrhage, *EDH* epidural hematoma, *ICU-LOS* intensive care unit length of stay, *H-LOS* hospital length of stay, *PTCI* posttraumatic cerebral infarction

**Table 2:** Outcomes data

Variables	PTCI (*n* = 47; 32.9%)	Non-PTCI (*n* = 96; 67.1%)	*p* value
Days of mechanical ventilation, mean (SD)	16.0 (9.3)	14.3 (10.4)	0.339
ICU-LOS, mean (SD)	21.8 (14.5)	19.9 (11.7)	0.042
H-LOS, mean (SD)	43.1 (46.2)	45.7 (40.6)	0.735
Primary outcome, 6-month GOS, no. (%)			
Good recovery—5	6 (13.0%)	36 (37.9%)	< 0.0001
Moderate disability—4	9 (19.6%)	32 (33.7%)	
Severe disability—3	15 (32.6%)	16 (16.8%)	
Vegetative state—2	3 (6.5%)	4 (4.2%)	
Death—1	13 (28.3%)	7 (7.4%)	
Secondary outcomes			
ICU mortality, no. (%)	10 (21.3%)	1 (1.0%)	< 0.0001
H mortality, no. (%)	12 (25.5%)	5 (5.2%)	0.980
6-month mortality, no. (%)	13 (27.7%)	7 (7.3%)	< 0.0001

Abbreviation: *ICU-LOS* intensive care unit length of stay, *H-LOS* hospital length of stay, *PTCI* posttraumatic cerebral infarction, *GOS* Glasgow Outcome Scale

After the central revision of the entire brain CT dataset, we correctly identified 10 patients with PTCI, missed at the initial evaluation; conversely, 7 patients initially classified as PTCI were subsequently classified as non-PTCI.

**Fig. 3:**
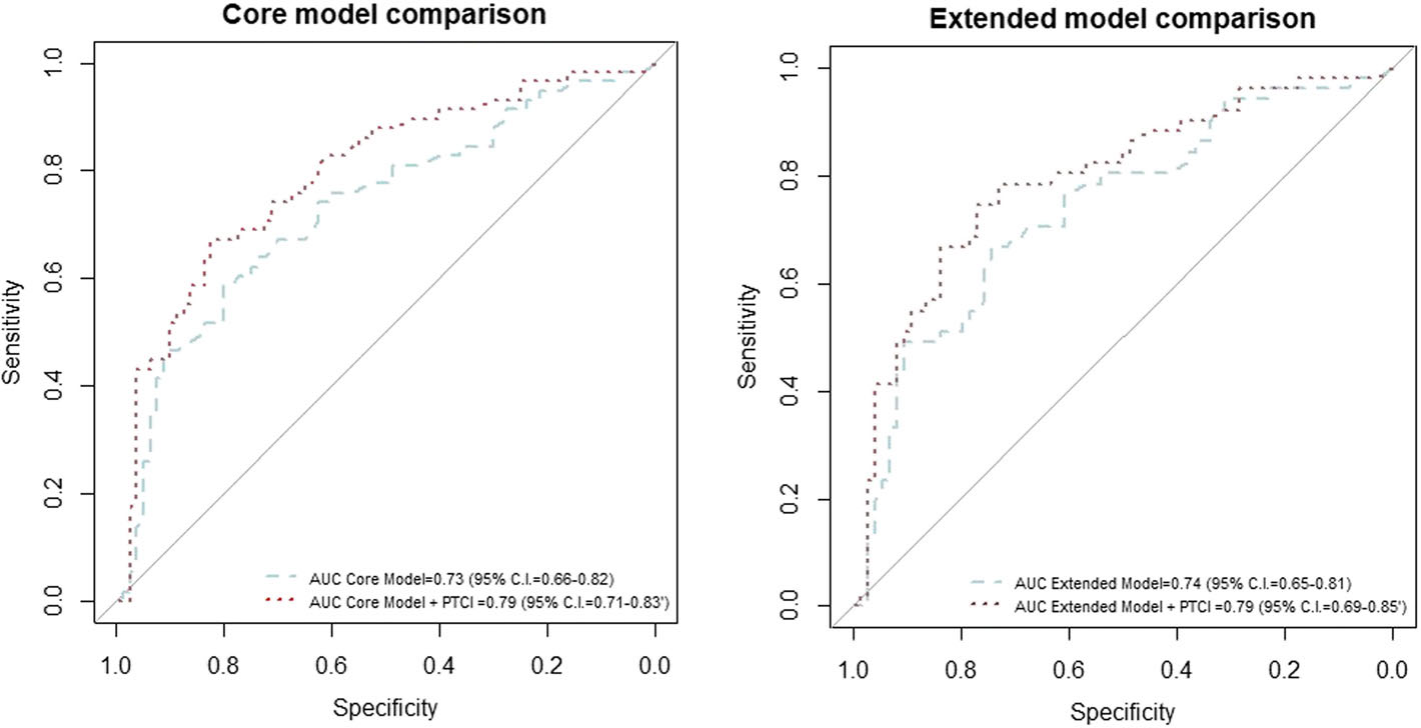
ROC curves for the core and extended IMPACT models with the addition of PTCI. Comparison of ROC curves and AUCs with and without the addition of PTCI, for both the core and extended models (*p* values for the difference in AUC: *p* = 0.05 for core model, *p* = 0.049 for the extended model). IMPACT: International Mission on Prognosis Analysis of Clinical Trials in Traumatic Brain Injury

**Fig. 4:**
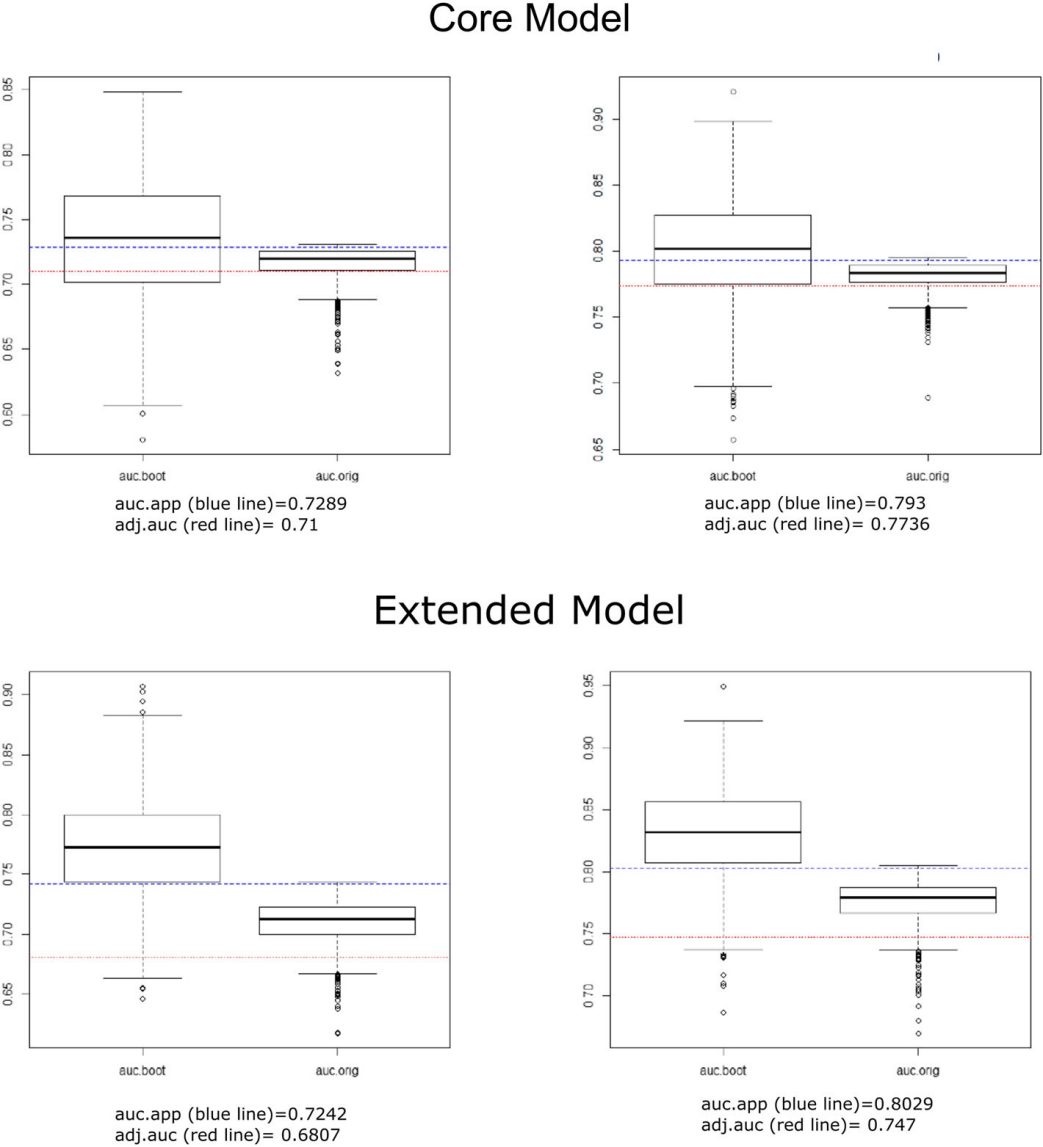
Correction for optimism of AUC for both core and extended models. Auc.boot is the distribution of the AUC value in the bootstrap sample, which represents “an estimation of the apparent performance.” “auc.orig” is the distribution of the AUC value deriving from the model fitted to the bootstrap samples and evaluated on the original sample, which represents the model performance on independent data. At the bottom of the chart, the apparent AUC (i.e., the value deriving from the model fitted to the original dataset) and the AUC adjusted for optimism are reported on the box plot respectively with the blue line and red line [25]

In the simple ordered logistic regression, the GOS was significantly worse in patients with a PTCI than patients without a PTCI, with a higher proportion of patients with a severe disability and death and a lower proportion of patients with good recovery and moderate disability (Table 2). In the multiple ordered logistic regression, a PTCI was retained in both the core and the extended models (Table 3a, b) as an independent predictor of the GOS. The predictive performances of the obtained models (Fig. 3) were good and increased significantly when PTCI was added to the IMPACT core model (AUC = 0.73, 95% C.I. 0.66–0.82 increased to AUC = 0.79, 95% CI 0.71–0.83; 0.0007) and the extended model (AUC = 0.74, 95% C.I. 0.65–0.81 increased to AUC = 0.80, 95% C.I. 0.69–0.85; *p* = 0.00008). The results were replicated after correcting the AUC for optimism (Fig. 4 and Table 4). Patients with a PTCI showed higher ICU mortality (10 patients [21.3%] vs one patient [1.0%], *p* < 0.0001) as well as higher 6-month mortality (13 patients [27.7%] vs 7 patients [7.3%], *p* < 0.0001); whereas hospital mortality did not differ between the two groups (Table 2).

**Table 3:** Adjusted ordered logistic regression for the Glasgow Outcome Scale (GOS). Each panel (A and B) includes the IMPACT model with the relative OR on the left (the core model in panel A and the extended model in panel B), and the recalculated OR when PTCI was added as a covariate on the right

Panel A	IMPACT core model			IMPACT core model plus PTCI		
GOS	OR	95% C.I.	*p*	OR	95% C.I.	*p*
Age	1.04	1.02–1.06	< 0.0001	1.04	1.02–1.06	< 0.0001
GCSm	0.43	0.22–0.84	0.011	0.45	0.23–0.90	0.025
Pupils	3.00	1.29–7.14	0.048	1.91	0.79–4.67	0.150
PTCI	–	–	–	3.88	1.85–8.34	< 0.0001
Panel B	IMPACT extended model			IMPACT extended model plus PTCI		
GOS	OR	95% C.I.	*p*	OR	95% C.I.	*p*
Age	1.04	1.01–1.06	< 0.0001	1.04	1.02–1.06	< 0.0001
GCSm	0.43	0.21–0.84	0.016	0.46	0.22–0.91	0.025
Pupils	3.32	1.33–8.51	0.031	2.26	0.89–5.82	0.085
Hypotension	1.60	0.51–3.78	0.310	0.52	0.78–4.99	0.147
Hypoxia	1.41	0.26–1.89	0.473	1.55	0.56–4.23	0.389
SAH	1.23	0.59–2.58	0.555	1.17	0.56–2.49	0.689
EDH	0.93	0.44–1.97	0.920	0.83	0.12–131.36	0.647
Marshall CT score	2.76	0.06–66.33	0.720	4.42	0.01–8.31	0.385
PTCI	–	–	–	4.77	2.19–10.67	< 0.0001

*GCSm* Glasgow Coma Scale motor score, *OR* proportional odds ratio, *C.I.* confidence interval, *SAH* traumatic subarachnoid hemorrhage, *EDH* epidural hematoma. Hypotension and hypoxia refer to both pre-hospital and intra-hospital periods

**Table 4:** Adjusted logistic regression for dichotomized GOS (favorable outcome: GOS = 4 and 5, and unfavorable outcome: GOS < 4) for core model and extended model with and without the addition of PTCI. AUC and AIC for each model are represented along with the ANOVA comparison between the model with and without the addition of PTCI. We used Nahelkerke’s Pseudo *R*^2^ for consistency with the original IMPACT study

	Core model			Extended model		
	OR	95% C.I.	*p*	OR	95 C.I.	*p*
Age	1.03	1.01–1.06	0.002	1.01	1.01–1.07	0.002
GCSm	0.34	0.14–0.77	0.012	0.33	1.27–7.14	0.015
Pupils	2.76	1.08–7.35	0.035	1.79	0.64–5.05	0.263
PTCI				4.52	1.88–11.39	< 0.001
AUC	0.73 (95% C.I.: 0.66–0.82)			0.79 (95% C.I.: 0.71–0.83)		
AUC comparison	Model with PTCI is better than the model without PTCI *p* = 0.0007					
Nagelkerke’s R^2^	0.21			0.33		
Age	1.03	1.01–1.06	0.003	1.03	1.01–1.06	0.004
GCSm	0.29	0.12–0.07	0.007	0.27	0.10–0.67	0.007
Pupils	3.11	1.14–8.90	0.027	2.09	0.68–6.48	0.190
Hypotension	2.24	0.73–7.12	0.160	3.29	0.99–11.60	0.055
Hypoxia	1.58	0.41–6.01	0.495	2.00	0.43–11.63	0.37
SAH	1.23	0.49–3.19	0.652	1.30	0.46–3.80	0.62
EDH	0.93	0.28–0.41	0.882	7.96	0.26–2.28	0.68
Marshall CT score	–	–	0.991	–	–	0.99
PTCI				6.84	2.58–19.7	< 0.001
AUC	0.74 (0.65–0.81)			0.82 (0.69–0.85)		
Nagelkerke’s R^2^	0.26			0.39		
AUC comparison	Model with PTCI is better than the model without PTCI *p* = 0.00008					

*GCSm* Glasgow Coma Scale motor score, *OR* proportional odds ratio, C.I. confidence interval, *SAH* traumatic subarachnoid hemorrhage, *EDH* epidural hematoma. Hypotension and hypoxia refer to both pre-hospital and intra-hospital periods

## Discussion

In this multicenter prospective cohort study, we found that a PTCI is an independent predictor of an unfavorable 6-month outcome and its addition to the IMPACT core and extended models increased their performance in predicting the GOS. Moreover, we confirmed that PTCI is a frequent complication occurring in more than one-third of patients suffering severe or moderate TBI. Most of the PTCIs were territorial, affecting one or more cerebral artery territories, and developed early during the ICU stay.

This is the first prospective study showing a PTCI has an independent effect on a patient’s long-term outcome. Previous studies were either post-mortem neuropathological investigations or ante-mortem retrospective clinical investigations. Among the latter, the GOS was assessed in four single-center studies at 3 months [5] or 6 months [4, 9]; while in one study, the timing of the GOS was not reported [8]. These studies showed increased morbidity [4], increased mortality [9], increased morbidity and mortality [8], or no difference [5] in patients with a PTCI compared with patients without. In three of these studies [4, 5, 9], the impact of a PTCI was assessed while considering the role of other predicting variables, such as age and GCS, using multiple regression analysis. However, none of these studies demonstrated that a PTCI adds value to risk prediction models that include validated factors, as we showed here. We added PTCI to the core and extended IMPACT models, which have been extensively validated with various datasets. The IMPACT models focus on baseline prognostic factors and do not include variables that develop throughout the disease process [3]. Therefore, our research expands the results of IMPACT, showing that patients developing a PTCI during the acute stage of disease have a five-fold increased risk for a poor outcome, independently from important factors such as age, motor score, pupillary reactivity, hypotension and hypoxia, brain CT, and the presence of posttraumatic SAH or EDH.

The AUC increased significantly from 0.73 to 0.79 and from 0.74 to 0.80 when the PTCI was added to the core and extended IMPACT models, respectively; although, these are already strong predictive models. Since the increase in the AUC greatly depends on the strength of the baseline model, the stronger the baseline model, the lesser the expected increase in the AUC [26]. This result further confirms that a PTCI is an important predictor of outcome in patients with a moderate or severe TBI. Our finding that a PTCI is a key independent predictor of long-term morbidity in TBI survivors is highly clinically plausible. Residual morbidity in patients suffering from cerebral infarction is high, with 13% of survivors discharged to institutional care [27]. Almost half of all elderly people suffering from an ischemic stroke have hemiparesis and cognitive impairment causing moderate to severe disability [28]. With severe stroke requiring ICU admission and mechanical ventilation, as many as two-thirds of surviving patients are left with a severe persisting disability [29]. The ICU mortality was significantly higher in patients with a PTCI, suggesting that a PTCI might be a proxy of the severity of the TBI; however, a multivariable analysis could not be performed due to the small number of deaths.

We found that PTCI was more frequent than in our previous retrospective study (32.9% vs 19.1%) [4]. In prospective cohort studies, the selection of patients through application of the inclusion and exclusion criteria is more accurate compared to retrospective studies, as it is the measurement of exposures before the outcome occurs, thereby establishing temporality and outcome. The availability of newer brain CT scanners, with higher sensitivity for detecting brain ischemia, may have also played a role. Not least, the centralized revision of all brain CTs increased the detection of PTCI, as 10 more PTCI cases were identified compared to diagnoses made locally by participating centers. This happened without sacrificing specificity because 7 patients initially classified as PTCI were subsequently classified as non-PTCI after centralized revision.

Our study has strengths and limitations. Strengths include the prospective multicenter nature of the study with central readings of all brain CT scans, as well as rigorous statistical methodology.

The identification of PTCI in ambiguous cases was greatly aided by the centralized evaluation of the neuroimaging data, allowing the assessment of the entire neuroradiological history of each patient, with immediate comparison of any questionable finding in a given CT scan to several previous and following exams, thus making the final diagnosis of cerebral infarction much more reliable.

The main limitation is the absence of detailed neurological and neuropsychological assessments of patients at long-term follow-up, limiting our understanding of the relative contribution of the primary traumatic and secondary ischemic brain damage to the persisting disability. Another limitation is that we did not assess the causes of mortality, and hence, we cannot exclude that life-sustaining therapies were withdrawn in patients with PTCI, leading to a self-fulfilling prophecy bias of the outcome prediction model. Moreover, results became available to the participating centers only after the entire brain CT data set of each patient was transferred to the coordinating center where the diagnosis of PTCI was definitely determined.

Our study is the first multicenter study demonstrating that a cerebral infarction, indicating posttraumatic brain damage, is an independent predictor of long-term disability when added to validated prediction models. This is in line with the recommendation of the Lancet Neurology Commission that prognostic models for TBI patients should include dynamic predictors that develop during the disease course [1]. Future research should externally validate this finding in larger studies with adequate power and accurate neurological and neuropsychological assessments of patients at longer-term follow-up. This would assess its generalizability and recommend the inclusion of PTCI in the list of measurable, clinically relevant variables that improve prognostication and contribute to a comprehensive definition of the diversity of post-TBI disability and the needs for personalized rehabilitation. The global burden of TBI has continuously increased in the last 25 years [30], and the prevention of residual disability is a major concern. Future studies should prioritize at risk-patients identification, along with effective prevention strategies to be deployed before the cerebral infarction is fully established.

## Conclusions

The findings provide evidence that PTCI is a common complication in patients suffering from a moderate or severe TBI and is an independent risk factor for long-term disability. The addition of PTCI to the IMPACT core and extended predictive models significantly increased their performance in predicting the GOS.

## Data Availability

Dataset of the present paper and R Code are available at Piva, Simone (2019), “Neurology-INCEPT Study Dataset”, Mendeley Data, https://data.mendeley.com/datasets/publish-confirmation/62thth3yyd/1

## Abbreviations

PTCI: Posttraumatic cerebral infarction
TBI: Traumatic brain injury
ICUs: Intensive care units
GOS: Glasgow Outcome Scale
IMPACT: International Mission for Prognosis and Clinical Trial design in TBI
GCSm: Glasgow Coma Scale motor score
CT: Computed tomography
DICOM: Digital Imaging and Communications in Medicine
SAH: Subarachnoid hemorrhage
EDH: Epidural hematoma
ACA: Anterior cerebral artery
MCA: Middle cerebral artery
PCA: Posterior cerebral artery
LSAs: Lenticulostriate arteries
TPAs: Thalamo-perforating arteries
BA: Basilar artery
AICA: Anterior–inferior cerebellar artery
SCA: Superior cerebellar artery
PICA: Posterior–inferior cerebellar artery
LOS: Length of stay
AUC: Receiver operating characteristic curve
ICP: Intracranial pressure
